# Avascular necrosis of the femoral head due to the bilateral injection of heroin into the femoral vein: A case report

**DOI:** 10.3892/etm.2013.1236

**Published:** 2013-07-26

**Authors:** DENGKE WU, DEYE SONG, JIANGDONG NI, RUCHUN DAI

**Affiliations:** 1 Department of Orthopedics, The Second Xiangya Hospital, Central South University, Changsha, Hunan 410011, P.R. China; 2 Institute of Metabolism and Endocrinology, The Second Xiangya Hospital, Central South University, Changsha, Hunan 410011, P.R. China

**Keywords:** avascular necrosis of the femoral head, intravenous injection, heroin

## Abstract

In this study, we report a case of avascular necrosis of the femoral head (ANFH) caused by the bilateral intravenous injection of heroin into the femoral vein. The patient had not used steroids, did not smoke and did not drink excessively. The patient did not present with any systemic diseases that may cause ANFH. ANFH often causes changes to the bone mass of the femoral head, particularly, the weight-bearing bone region. Imaging examination revealed that in addition to the bilateral hip joints, widespread changes to the bone mass existed in the peripheral area, where phlebitis and disseminated thrombosis had been caused by the injection of heroin. These results suggest that this ANFH case was related to the injection of heroin. This case is different from other cases of ANFH which have been caused by steroids and other factors, including smoking, alcohol intake and cytotoxic agents.

## Introduction

Avascular necrosis of the femoral head (ANFH) is caused by disruption of the blood supply to the bone, followed by hip dysfunction (1). Femoral head ischemia results in the death of marrow and osteocytes and the necrotic segment subsequently collapses (2). X-ray images of ANFH patients in their early stages usually appear normal. However, in the later stages, the images often appear increasingly radio-opaque. The necrotic bones themselves do not demonstrate increases in radio-graphic opacity, since dead bones do not undergo the bone resorption usually performed by living osteoclasts. However, the detailed mechanism for the pathogenesis of non-traumatic ANFH in young patients is unclear. The common causes of ANFH include short-term use of a large quantity of steroids and excessive drinking. Certain diseases may also result in ANFH, such as systemic lupus erythematous (3), hemoglobin-opathies (4,5), Legg-Calvé-Perthes disease, Gaucher’s disease, dysbarism, HIV, hyperlipidemia, pancreatitis and gout (6,7). Exposure to radiation or cytotoxic agents may also result in ANFH (8,9). However, ANFH caused by heroin injection is rarely reported. In the current study, we report a rare case of ANFH caused by the bilateral injection of heroin into the femoral vein.

## Case report

**Figure 1.**
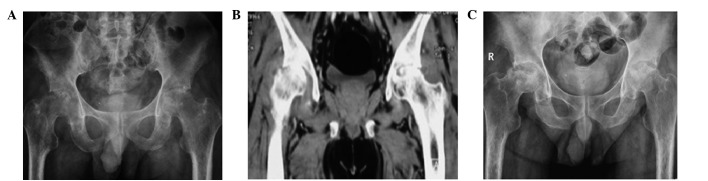
X-ray of the pelvis reveals bilateral femoral head collapse. (A) Widespread changes to the bone mass of the pelvis and bilateral femurs. The bilateral femoral head is flat, with an uneven bone density. A region of multiple small cystic bone destruction under the articular surface is visible, with a rough articular surface. The joint space is narrowed, the upper edge of the acetabular is hardened and the right lateral femoral head has shifted slightly outwards to the eutopic left hip. (B) Computed tomography shows ischemic necrosis of the bilateral femoral head and effusion of the right hip joint. The surrounding tissue of the bilateral hip is swollen. The load-bearing surface joint space of the right hip joint has narrowed, whereas the non-load-bearing surface joint space has widened. The gap in the left hip is uniformly narrower, the head shape of the bilateral femor is irregularly flat, the surface is rough, multiple bone density-reducing regions are visible in the bone mass and the surrounding soft tissue is swollen. (C) Pelvic X-ray after six months of treatment reveals that the collapse of the bilateral femoral head of the patient is more severe than that six months earlier. The bilateral femoral head was flatter, which indicates that the ANFH of the patient had developed gradually following the injection of heroin.

A 38-year-old male patient presented with bilateral hip pain that had been occurring for five months. The patient had no history of smoking or excessive drinking. Prior written and informed consent were obtained from the patient and the study was approved by the ethics review board of The Second Xiangya Hospital, Central South University, (Changsha, China). The patient had smoked heroin for 13 years and had bilaterally injected heroin into the femoral vein for two years. Physical examination indicated a 160° flexion deformity at the bilateral hip joints and point tenderness in the bilateral inguinal midpoint, but no significant swelling or skin pigmentation. Routine blood test results of liver and kidney electrolytes were normal. Pelvic X-ray examination showed ischemic necrosis of the bilateral femoral head, subluxation of the right hip and osteoporosis of the pelvis and bilateral femurs (Fig. 1A). A computed tomography (CT) scan of the hip joint indicated ischemic necrosis of the bilateral femoral head, detectible effusion in the right hip joint and soft tissue swelling in the bilateral hip (Fig. 1B). Vascular color Doppler ultrasound revealed double lower limb phlebitis and disseminated thrombosis. The single-photon emission computed tomography (SPECT) results indicated that the metabolism of the bilateral hip joint was active. The electrocardiogram (ECG) and chest X-ray results were normal.

The patient was treated with Aescuven^®^ forte (Cesra Arzneimittel GmbH & Co. KG, Baden-Baden, German) 300 mg orally, twice a day, alfacalcidol (Chemvon Biotechnology Co., Ltd., Shanghai, China) 0.5 *μ*g orally, once a day, and one D-Cal^®^ tablet (300 mg of Calcium and 100 IU of Vitamin D3; A&Z Pharmaceutical Inc., New York, NY, USA) orally, twice a day. The patient stopped injecting heroin during the treatment. Following six months of treatment, the bilateral hip pain of the patient was reduced. Physical examination indicated a reduction in bilateral inguinal tenderness compared with that exhibited six months previously. The self-movement of the hip had also improved and the hip was now capable of bending from 180 to 140°. However, the bilateral hip extension, adduction, abduction, rotation and other functions remained limited. The blood sedimentation, C-reactive protein level and blood coagulation function returned to normal levels. An X-ray of the pelvis revealed that the bilateral femoral head of the patient had collapsed (Fig. 1C). Vascular color Doppler ultrasound revealed thromboses in the veins in the patient’s lower limbs.

## Discussion

ANFH caused by the intravenous injection of heroin is rare, but there are many cases of thrombophlebitis resulting from the intravenous injection of heroin (10,11). Pieper and Templin observed that chronic venous insufficiency is a common symptom in patients who have injected drugs (12). The femoral intravenous injection of heroin promotes platelet adhesion and fibrin deposition, leading to venous thrombosis (6,7,13). Venous thrombosis leads to increased intraosseus venous pressure and reduced arterial flow, which results in hypoxic bone death (6,13). The inflammation and thrombosis of the venous system of the hip due to the long-term intravenous injection of heroin demonstrates a marked correlation with ANFH and the destruction of the surrounding bone (14,15). Our results suggest that the long-term injection of heroin into the femoral vein of the patient leads to increased blood viscosity, phlebitis and disseminated thrombosis, which inhibits the blood circulation to the femoral head, leading to ischemic necrosis of the femoral head (17–19).

The efficacy of drug treatment for ANFH is usually unsatisfactory. In the current case, following admission, the patient received therapy to improve the blood circulation to the femoral head and to facilitate the formation of new bone. As the blood supply to the ischemic femoral head improved, the pain decreased. However, the formation of new bone is a slow process. Furthermore, the patient did not carefully follow the recommendations of the doctor and performed weight-bearing exercises, which led to the further collapse of the femoral head.
